# Angiogenic Imbalance Defines Multisystem Phenotypes of Preeclampsia: A Phenotype-Oriented Cohort Study

**DOI:** 10.3390/clinpract16040076

**Published:** 2026-04-17

**Authors:** Anca Tătaru-Copos, Florin Szasz, Anca Carmen Huniadi, Rodica Georgeta Negrini, Mircea Ioachim Popescu, Paula Trif, Gelu Florin Murvai, Radu Galiș, Cristian Sava, Romina Viorela Murvai

**Affiliations:** 1 Doctoral School of Biological and Biomedical Sciences, University of Oradea, 1 University Street, 410087 Oradea, Romania; 2 Department of Surgical Sciences, Obstetrics and Gynecology, Faculty of Medicine and Pharmacy, University of Oradea, 1 University Street, 410087 Oradea, Romania; 3 Department of Obstetrics and Gynecology, Emergency County Hospital Bihor, 65 Gheorghe Doja Street, 410169 Oradea, Romania; 4 Calla—Infertility Diagnostic and Treatment Center, Constantin A. Rosetti Street, 410103 Oradea, Romania; 5 Pelican Clinical Hospital, Corneliu Coposu Street 2, 410450 Oradea, Romania; 6 Department of Cardiology, Emergency County Hospital Bihor, 65 Gheorghe Doja Street, 410169 Oradea, Romania; 7 Department of Cardiology, Faculty of Medicine and Pharmacy, University of Oradea, 1 University Street, 410087 Oradea, Romania; 8 Department of Orthopedics and Traumatology I, Bihor County Emergency Clinical Hospital, 65 Gheorghe Doja Street, 410169 Oradea, Romania; 9 Department of Neonatology, Emergency County Hospital Bihor, 410167 Oradea, Romania; 10 Department of Medical Sciences, Faculty of Medicine and Pharmacy, University of Oradea, 410087 Oradea, Romania; 11 Department of Pediatrics, Emergency County Hospital Bihor, 410167 Oradea, Romania

**Keywords:** preeclampsia, sFlt-1/PlGF ratio, angiogenic imbalance, phenotyping, multisystem involvement, perinatal outcomes, endothelial dysfunction, precision obstetrics

## Abstract

Background: Preeclampsia is a heterogeneous multisystem disorder characterized by endothelial dysfunction and angiogenic imbalance. While the sFlt-1/PlGF ratio is widely used for diagnostic purposes, its role in defining biological phenotypes of preeclampsia remains insufficiently explored. This study aimed to investigate whether angiogenic imbalance is associated with distinct multisystem phenotypes of preeclampsia and with perinatal outcomes. Methods: We conducted a retrospective cohort study including 320 pregnant women, of whom 68 were diagnosed with preeclampsia. Multisystem phenotypes were defined using laboratory markers reflecting renal, hepatic, and hematologic involvement. The sFlt-1/PlGF ratio was compared across phenotypes. Associations with gestational age at delivery, birth weight, Apgar score, and neonatal intensive care unit (NICU) admission were evaluated. Receiver operating characteristic (ROC) analysis assessed the discriminatory performance of the sFlt-1/PlGF ratio for identifying the renal-dominant phenotype. Results: The mean sFlt-1/PlGF ratio was higher in preeclampsia compared to normotensive pregnancies (58.5 ± 20.3 vs. 34.6 ± 15.9). Within preeclampsia, the renal-dominant phenotype showed the highest ratio (66.0 ± 22.5), followed by hepatic (55.9 ± 18.2) and hematologic phenotypes (52.0 ± 16.8). The renal phenotype was associated with earlier delivery (34.6 weeks), lower birth weight (2196 g), higher NICU admission (10.7%), and lower Apgar scores. The sFlt-1/PlGF ratio demonstrated moderate discrimination for the renal phenotype (AUC = 0.69). Conclusions: Angiogenic imbalance varies across multisystem phenotypes of preeclampsia and is associated with meaningful perinatal differences. The sFlt-1/PlGF ratio may contribute to phenotype-based risk stratification, supporting a move toward precision obstetrics. Prospective studies are needed to validate phenotype-oriented classification models.

## 1. Introduction

Preeclampsia (PE) remains one of the leading causes of maternal and perinatal morbidity and mortality worldwide, affecting approximately 3–8% of pregnancies. Traditionally defined by new-onset hypertension and proteinuria after 20 weeks of gestation, preeclampsia is now recognized as a complex, multisystem disorder involving widespread endothelial dysfunction, immune dysregulation, and placental maladaptation. Its clinical expression is highly heterogeneous, ranging from mild forms with minimal organ involvement to severe disease with renal, hepatic, hematologic, and neurological complications [1,2,3].

Over the past two decades, increasing attention has been directed toward the role of angiogenic imbalance in the pathophysiology of preeclampsia. The dysregulated production of anti-angiogenic factors, particularly soluble fms-like tyrosine kinase-1 (sFlt-1), and the concomitant reduction in pro-angiogenic placental growth factor (PlGF) contribute to systemic endothelial dysfunction and impaired maternal vascular adaptation. The sFlt-1/PlGF ratio has consequently emerged as a valuable biomarker for the diagnosis and short-term prediction of preeclampsia, and it is increasingly integrated into clinical decision-making algorithms [4,5,6].

However, most studies have approached the sFlt-1/PlGF ratio primarily as a diagnostic or prognostic tool, rather than as a marker reflecting the biological heterogeneity of the disease. Preeclampsia is increasingly viewed not as a single entity but as a syndrome encompassing multiple pathophysiological pathways and clinical phenotypes. Some patients exhibit predominant renal involvement, others show hepatic or hematologic disturbances, while a subset presents with early-onset placental dysfunction and fetal growth restriction. This variability suggests that different degrees or patterns of angiogenic imbalance may be associated with distinct multisystem phenotypes [7,8,9,10,11].

A phenotype-oriented approach may therefore provide a more nuanced understanding of preeclampsia by linking angiogenic status to patterns of maternal organ involvement and perinatal risk. Despite this conceptual shift, data integrating angiogenic markers with multisystem clinical phenotyping remain limited [12,13].

Although previous studies have established the diagnostic and short-term prognostic utility of the sFlt-1/PlGF ratio, most recent literature from the past 3–5 years has primarily focused on binary prediction models for preeclampsia or on fetal growth restriction and placental insufficiency rather than on phenotype-oriented maternal multisystem stratification. Recent reports have emphasized the role of angiogenic markers in identifying placental dysfunction and adverse fetal outcomes, but data linking these biomarkers to distinct maternal organ-dominant phenotypes remain limited. Therefore, an important research gap persists regarding whether angiogenic imbalance may help biologically classify preeclampsia beyond conventional diagnostic thresholds.

In this context, the present study aimed to investigate whether angiogenic imbalance, assessed by the sFlt-1/PlGF ratio, is associated with distinct multisystem phenotypes of preeclampsia and whether these phenotypes are linked to adverse perinatal outcomes.

## 2. Materials and Methods

### 2.1. Study Design and Population

This retrospective observational cohort study was conducted on pregnant women who delivered at a tertiary care obstetrics center between [2019–2023]. Medical records were reviewed to identify women with available clinical, obstetric, and laboratory data, including angiogenic biomarkers. Pregnancy plurality was not systematically recorded and therefore could not be included in the analysis.

A total of 320 pregnant women were included in the final analysis. Among them, 68 women were diagnosed with preeclampsia (PE), while 252 women without preeclampsia served as the comparison group. A total of 320 pregnant women were included in the final analysis. Among them, 68 women were diagnosed with preeclampsia (PE), while 252 women without preeclampsia served as the comparison group. Diagnostic criteria are detailed in Section 2.4.

Women with incomplete data on angiogenic biomarkers or key outcome variables were excluded from the analysis.

### 2.2. Clinical and Obstetric Data Collection

Maternal demographic and clinical characteristics collected included maternal age and place of residence (urban/rural). Obstetric variables comprised gestational age at delivery, mode of delivery, length of hospitalization, and the presence of pregnancy-related comorbidities, including gestational diabetes and gestational hypertension (distinct from preeclampsia).

Neonatal outcomes assessed were birth weight, Apgar (Appearance, Pulse, Grimace, Activity, and Respiration) scores at 1 and 5 min, sex of the newborn, and admission to the neonatal intensive care unit (NICU). Only NICU admission (yes/no) was available; NICU length of stay was not consistently recorded. Gestational age at biomarker sampling was not consistently recorded.

Maternal complications included clinically documented obstetric complications recorded in medical records (e.g., postpartum hemorrhage, hypertensive complications, or other significant peripartum events).

Severity stratification was not feasible due to inconsistent documentation.

### 2.3. Measurement of Angiogenic Biomarkers

Maternal serum levels of soluble fms-like tyrosine kinase-1 (sFlt-1) and placental growth factor (PlGF) were measured as part of routine clinical evaluation for suspected hypertensive disorders of pregnancy. The sFlt-1/PlGF ratio was calculated by dividing the serum concentration of sFlt-1 by that of PlGF.

All measurements were performed prior to delivery. All biomarker measurements were performed using standardized, clinically validated immunoassays in the hospital laboratory, in accordance with the manufacturer’s instructions. Blood sampling was performed at the time of clinical evaluation for suspected hypertensive disorders of pregnancy in all participants. The non-preeclamptic group included women in whom preeclampsia was clinically suspected but subsequently ruled out.

Serum sFlt-1 and PlGF concentrations were measured using automated electro-chemiluminescence immunoassays (ECLIA) on the Roche Cobas e601 platform (Roche Diagnostics, Mannheim, Germany), according to the manufacturer’s instructions. The Elecsys sFlt-1 and Elecsys PlGF kits were used. All assays were performed in the same laboratory, and internal quality controls were used according to manufacturer recommendations.

No formal multiplicity correction was applied; findings should be interpreted cautiously.

### 2.4. Outcome Definitions

The primary outcome of interest was the presence of preeclampsia. Preeclampsia was defined according to the American College of Obstetricians and Gynecologists (ACOG) criteria as new-onset hypertension (systolic blood pressure ≥ 140 mmHg and/or diastolic blood pressure ≥ 90 mmHg on two occasions at least 4 h apart after 20 weeks of gestation), associated with proteinuria and/or evidence of maternal end-organ dysfunction.

Secondary outcomes included:

- Gestational age at delivery (weeks);
- Birth weight (grams);
- Apgar scores at 1 and 5 min;
- Length of maternal hospitalization (days);
- NICU admission (yes/no).

### 2.5. Statistical Analysis

Phenotype definition (preeclampsia subgroup): Multisystem phenotypes were assigned within the preeclampsia group using standardized (z-score) laboratory markers. A renal involvement score was computed as z(creatinine) + z(uric acid) + z(proteinuria); a hepatic score as z(AST) + z(ALT); and a hematologic score as -z(platelets) (lower platelet counts indicating greater involvement). Each patient was assigned to the phenotype corresponding to the highest score (renal-, hepatic-, or hematologic-dominant).

Statistical analyses were performed using IBM SPSS Statistics (version 30; IBM Corp., Armonk, NY, USA). Continuous variables were assessed for normality using visual inspection and distributional characteristics.

Continuous data are presented as median [interquartile range] or mean ± standard deviation, as appropriate. Categorical variables are presented as counts and percentages. Between-group comparisons were conducted using the Mann–Whitney U test or Welch’s *t*-test for continuous variables and the Chi-square test or Fisher’s exact test for categorical variables, as appropriate.

Because of the retrospective cohort design, no a priori sample size or power calculation was performed. All consecutive eligible patients meeting the inclusion criteria during the study period were included.

Given the exploratory nature of several analyses, findings should be interpreted cautiously due to potential type I error inflation.

The diagnostic performance of the sFlt-1/PlGF ratio for identifying preeclampsia was evaluated using receiver operating characteristic (ROC) curve analysis, with calculation of the area under the curve (AUC) and corresponding 95% confidence intervals (CI). The optimal cut-off value was determined using the Youden index. For all key effect estimates, 95% confidence intervals were calculated where applicable, including ROC area under the curve estimates.

Given the exploratory nature and limited subgroup sizes, formal multiplicity correction was not applied; therefore, subgroup comparisons should be interpreted as hypothesis-generating.

A two-sided *p*-value < 0.05 was considered statistically significant. The derived threshold should not replace established GA-specific cut-offs. The interval between biomarker sampling and delivery or neonatal outcomes was not consistently documented.

### 2.6. Ethical Considerations

The study was conducted in accordance with the Declaration of Helsinki. Ethical approval was obtained from the local institutional ethics committee. Due to the retrospective nature of the study and the use of anonymized data, the requirement for informed consent was waived.

## 3. Results

### 3.1. Demographic and Baseline Characteristics

A total of 320 pregnant women were included in the cohort, of whom 68 (21.3%) were diagnosed with preeclampsia and 252 (78.7%) had normotensive pregnancies.

The overall mean maternal age was 31.6 ± 5.9 years and did not differ between groups (31.1 ± 7.4 in preeclampsia vs. 31.8 ± 5.4 in normotensive pregnancies; *p* = 0.621).

Regarding obstetric history, 35.9% of the cohort were in their first pregnancy (Gesta = 1). First pregnancies were more frequent among women with preeclampsia (50.0%) compared to normotensive women (32.1%).

The mean gestational age at delivery was 37.6 ± 2.3 weeks overall and was lower in pregnancies with preeclampsia (35.8 ± 3.4 vs. 38.1 ± 1.6 weeks; *p* < 0.001).

Gestational diabetes was present in 3.8% of the total cohort and in 2.9% of women with preeclampsia. Gestational hypertension was recorded in 8.4% of cases overall and in 2.9% of women with preeclampsia.

Neonatal intensive care unit (NICU) admission was required in 2.2% of all deliveries and in 5.9% of pregnancies complicated by preeclampsia.

Baseline demographic and obstetric characteristics are summarized in Table 1.

### 3.2. Clinical and Laboratory Characteristics

The clinical and laboratory profiles of the study population are summarized in Table 2.

Women with preeclampsia showed differences in several laboratory parameters compared to normotensive pregnancies.

#### 3.2.1. Hematologic Parameters

The mean platelet count (PLT) in the overall cohort was 231.0 ± 63.7 × 10^3^/µL. Women with preeclampsia had slightly lower platelet levels (222.2 ± 68.9 × 10^3^/µL) compared to those without preeclampsia (233.4 ± 62.2 × 10^3^/µL).

The median white blood cell count (WBC) was 11.71 [9.85, 13.60] × 10^3^/µL overall, with similar values in the preeclampsia group (11.85 [9.65, 13.74]) and normotensive pregnancies (11.68 [9.87, 13.56]).

Fibrinogen levels were comparable between groups, with a mean value of 528.3 ± 119.3 mg/dL in the overall cohort.

#### 3.2.2. Hepatic Markers

Liver enzymes were higher in women with preeclampsia.

Mean AST level was 29.4 ± 49.3 U/L in preeclampsia vs. 20.6 ± 30.4 U/L in normotensive pregnancies.

Mean ALT level was 38.6 ± 74.7 U/L in preeclampsia vs. 18.7 ± 26.2 U/L in those without preeclampsia.

#### 3.2.3. Renal Markers

Renal function markers showed modest differences between groups.

Mean serum creatinine was 0.63 ± 0.06 mg/dL in preeclampsia and 0.62 ± 0.07 mg/dL in normotensive pregnancies.

Mean uric acid levels were slightly higher in preeclampsia (4.55 ± 1.08 mg/dL) compared to non-preeclamptic pregnancies (4.33 ± 1.12 mg/dL).

Proteinuria showed the most notable difference, with a markedly higher mean value in women with preeclampsia (99.4 ± 94.4) compared to those without preeclampsia (15.3 ± 27.5).

**Figure 1:**
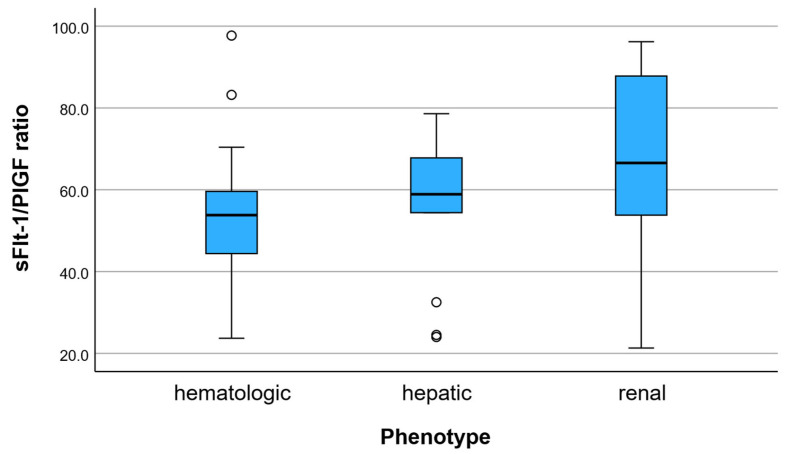
sFlt-1/PlGF ratio across multisystem preeclampsia phenotypes. Distribution of the sFlt-1/PlGF ratio according to dominant clinical phenotype. Phenotypes were defined based on the predominant laboratory involvement: renal (creatinine, uric acid, proteinuria), hepatic (AST, ALT), and hematologic (platelet count). The boxplots illustrate variability and median differences in angiogenic imbalance across phenotypes.

Overall, preeclampsia was associated with a laboratory profile suggestive of multisystem involvement, including hepatic, renal, and hematologic alterations (Figure 1).

### 3.3. Angiogenic Profile Results

The angiogenic profile of the cohort, assessed by the sFlt-1/PlGF ratio, showed marked differences between pregnancies complicated by preeclampsia and normotensive pregnancies.

The mean sFlt-1/PlGF ratio in the overall cohort was 39.7 ± 19.5. Women with preeclampsia exhibited substantially higher values, with a mean ratio of 58.5 ± 20.3, compared to 34.6 ± 15.9 in normotensive pregnancies.

The between-group difference in sFlt-1/PlGF ratio corresponded to a large standardized effect size (Cohen’s d = 1.31). In ROC analysis, the sFlt-1/PlGF ratio showed good discrimination for preeclampsia (AUC = 0.81 (95% CI: 0.76–0.84)). ROC analysis demonstrated moderate discrimination for the renal phenotype 0.69 (95% CI: 0.62–0.74, *p* = 0.012).

**Figure 2:**
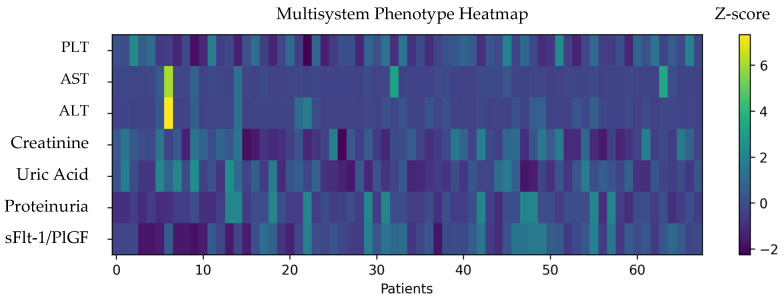
Multisystem phenotype heatmap in patients with preeclampsia. Heatmap showing z-score standardized laboratory parameters across all preeclampsia cases included in the phenotype analysis (*n* = 68). Each column represents one patient, and each row corresponds to a biological marker reflecting hematologic (PLT), hepatic (AST, ALT), renal (creatinine, uric acid, proteinuria), and angiogenic (sFlt-1/PlGF ratio) involvement. Higher z-score values indicate greater deviation from the cohort mean and are represented by warmer colors, while lower values are shown by cooler colors. This visualization highlights the heterogeneity of multisystem involvement and supports the phenotype-oriented classification framework.

Within the preeclampsia group, the sFlt-1/PlGF ratio varied across multisystem phenotypes (Figure 2). The renal-dominant phenotype showed the highest angiogenic imbalance, with a mean ratio of 66.0 ± 22.5. The hepatic phenotype presented intermediate values (55.9 ± 18.2), while the hematologic phenotype had comparatively lower values (52.0 ± 16.8).

A progressive increase in the sFlt-1/PlGF ratio was observed from hematologic to hepatic and renal phenotypes, indicating a gradient of angiogenic dysregulation across patterns of organ involvement.

These findings support the concept that angiogenic imbalance is not uniform in preeclampsia but varies according to the dominant pattern of multisystem involvement.

### 3.4. Association with Perinatal Outcomes

Perinatal outcomes differed across preeclampsia phenotypes, showing numerical differences across phenotypes, although not all comparisons reached statistical significance (Table 3).

#### 3.4.1. Gestational Age at Delivery

Mean gestational age at delivery was lowest in the renal-dominant phenotype (34.6 ± 3.9 weeks), followed by the hepatic phenotype (35.9 ± 2.8 weeks) and the hematologic phenotype (36.9 ± 2.8 weeks). This pattern indicates a higher burden of prematurity in the renal-dominant group.

#### 3.4.2. Birth Weight

Neonatal birth weight also varied across phenotypes. The lowest mean birth weight was observed in the renal phenotype (2196 ± 1011 g), compared to 2633 ± 812 g in the hepatic phenotype and 3007 ± 846 g in the hematologic phenotype.

#### 3.4.3. NICU Admission

NICU admission rates were highest in the renal-dominant phenotype (10.7%), followed by the hepatic phenotype (7.7%), while no NICU admissions were recorded in the hematologic phenotype.

#### 3.4.4. Apgar Score

Mean Apgar score at 1 min showed a decreasing trend from hematologic (8.0) to hepatic (7.8) and renal phenotypes (6.6), suggesting more compromised neonatal adaptation in cases with predominant renal involvement.

**Figure 3:**
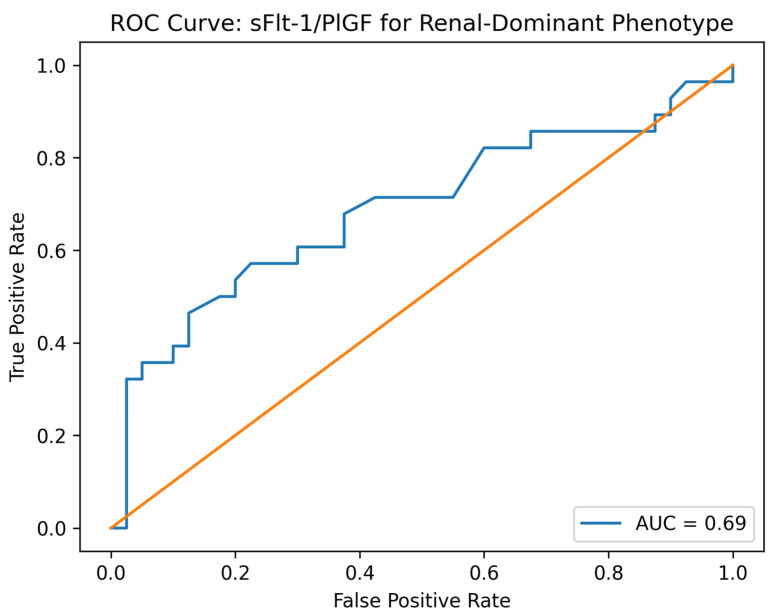
Receiver operating characteristic (ROC) curve for the identification of the renal-dominant phenotype using the sFlt-1/PlGF ratio. The ROC curve illustrates the discriminatory performance of the angiogenic ratio for distinguishing the renal-dominant phenotype from other phenotypes of preeclampsia. The area under the curve (AUC) was 0.69 (95% 0.62–0.74, *p* = 0.012), indicating moderate discriminatory ability. The associated *p*-value is shown in the figure.

Overall, the renal-dominant phenotype was associated with less favorable perinatal outcomes, including earlier delivery, lower birth weight, higher NICU admission rates, and lower Apgar scores (Figure 3).

## 4. Discussion

The present phenotype-oriented cohort study supports the concept that preeclampsia is a biologically heterogeneous syndrome rather than a single clinical entity. By integrating angiogenic markers with multisystem laboratory profiles, we identified distinct preeclampsia phenotypes and demonstrated that the degree of angiogenic imbalance varies across patterns of organ involvement and is associated with perinatal outcomes.

### 4.1. Angiogenic Imbalance as a Marker of Phenotypic Severity

One of the key findings of our study is that the sFlt-1/PlGF ratio differed across multisystem phenotypes, with the highest values observed in the renal-dominant phenotype. This supports the view that angiogenic imbalance is closely linked to the extent and type of maternal organ involvement. While the sFlt-1/PlGF ratio is widely used for diagnostic and short-term prediction purposes, our results suggest that it also reflects the biological severity and systemic expression of the disease [14,15,16]. The relatively moderate mean sFlt-1/PlGF ratio observed in the PE group may reflect the inclusion of predominantly later-onset or less severe forms of disease, as well as the heterogeneity inherent to retrospective real-world cohorts.

The observed gradient—from hematologic to hepatic to renal phenotypes—may indicate progressive endothelial and microvascular dysfunction. Renal involvement in preeclampsia is strongly dependent on endothelial injury and glomerular endotheliosis, mechanisms that are directly influenced by anti-angiogenic factors. Therefore, it is plausible that higher sFlt-1/PlGF ratios are particularly associated with renal-dominant disease expression.

### 4.2. Preeclampsia as a Multisystem and Multiphase Disorder

Our heatmap analysis illustrates the heterogeneity of laboratory alterations among women with preeclampsia, reinforcing the idea that different patients may follow partially distinct pathophysiological pathways. Some cases show predominant hepatic involvement, others hematologic changes, and others renal dysfunction. This variability aligns with emerging models describing preeclampsia as a syndrome with multiple endotypes rather than a uniform disorder.

Such heterogeneity may explain why clinical severity does not always correlate perfectly with traditional diagnostic thresholds. A phenotype-oriented framework may therefore offer a more nuanced understanding of disease progression and risk stratification [17,18,19,20].

### 4.3. Clinical Relevance for Perinatal Outcomes

The renal-dominant phenotype was consistently associated with less favorable perinatal outcomes, including earlier delivery, lower birth weight, higher NICU admission rates, and lower Apgar scores [21]. These findings support the concept that maternal endothelial and renal dysfunction are closely linked to placental insufficiency and fetal compromise [22,23,24,25].

Our findings are also consistent with recent evidence supporting the broader role of angiogenic biomarkers in placental dysfunction beyond classical preeclampsia definitions. Libretti et al. highlighted the relevance of the sFlt-1/PlGF ratio in pregnancies complicated by fetal growth restriction and small-for-gestational-age fetuses, emphasizing its value as a marker of placental insufficiency. This supports our observation that the renal-dominant phenotype, characterized by the highest angiogenic imbalance, was also associated with the most adverse perinatal outcomes [26,27,28].

Importantly, these associations suggest that phenotype stratification may have practical clinical implications. Identifying a renal-dominant pattern together with a high sFlt-1/PlGF ratio could help clinicians recognize pregnancies at higher risk for prematurity and neonatal complications, potentially informing surveillance and timing of delivery.

### 4.4. Discriminatory Performance of the sFlt-1/PlGF Ratio

The ROC analysis showed moderate performance (AUC = 0.69) for discriminating the renal-dominant phenotype. While this supports the ability of the sFlt-1/PlGF ratio to contribute to phenotype stratification, its discriminatory capacity is clearly lower than its performance for the diagnosis of preeclampsia overall (AUC = 0.81). Therefore, the ratio should not be considered a definitive standalone phenotyping tool, but rather a complementary biomarker that adds biological and prognostic information when interpreted alongside clinical and laboratory parameters [7,29,30,31].

### 4.5. Implications for Precision Obstetrics

Our findings align with the growing paradigm of precision obstetrics, which seeks to move beyond binary diagnostic categories toward biologically informed stratification. Rather than viewing preeclampsia solely as present or absent, a phenotype-based approach may allow more individualized monitoring and management. Angiogenic markers could thus serve not only as diagnostic tools but also as instruments for biological classification [20,32,33,34,35]. Such an approach aligns with the broader movement toward precision medicine in obstetrics, where biological markers are used not only for diagnosis but also for stratifying disease subtypes and guiding individualized management.

### 4.6. Limitations

This study has several limitations. First, its retrospective design limits causal inference. Second, phenotypes were defined using available laboratory parameters, which may not fully capture the complexity of organ involvement. Third, the overall number of patients with preeclampsia was relatively limited (*n* = 68), and subdivision into three phenotypic groups resulted in small subgroup sizes, particularly for the hepatic phenotype (*n* = 13). This may have reduced the statistical power to detect small or moderate differences between subgroups and increased the possibility of type II error. Therefore, subgroup comparisons should be interpreted with caution. Finally, longitudinal angiogenic measurements were not available, preventing trajectory analyses.

A major limitation is the lack of consistently recorded gestational age at biomarker sampling, which is known to significantly influence sFlt-1/PlGF values and may affect the comparability of intergroup analyses. Additionally, assigning each patient to a single dominant phenotype may oversimplify the multisystem nature of severe preeclampsia, in which overlapping organ involvement is common. Future studies should consider hybrid or multidimensional phenotype models.

Stratification into early-onset (<34 weeks) and late-onset (≥34 weeks) preeclampsia was not consistently feasible because the exact timing of disease onset was not uniformly documented.

Potential confounding factors, including parity, gestational diabetes, and gestational age at delivery, may have influenced the observed associations and could not be fully adjusted for because of the sample size and retrospective design.

### 4.7. Future Directions

Future prospective studies with larger cohorts and repeated angiogenic measurements are needed to validate phenotype-based classification models. Integrating imaging, placental biomarkers, and molecular profiling could further refine the identification of preeclampsia endotypes. Such approaches may ultimately support personalized surveillance strategies and optimized timing of delivery.

## 5. Conclusions

This phenotype-oriented cohort study highlights the heterogeneity of preeclampsia and supports the concept that angiogenic imbalance is differentially expressed across multisystem phenotypes. The sFlt-1/PlGF ratio was highest in the renal-dominant phenotype and followed a gradient across hepatic and hematologic patterns, suggesting that angiogenic dysregulation reflects the dominant pathway of organ involvement.

Importantly, phenotypic stratification was clinically meaningful, as the renal-dominant phenotype was associated with earlier delivery, lower birth weight, higher NICU admission rates, and lower Apgar scores. These findings indicate that integrating angiogenic markers with multisystem clinical profiles may contribute to improved risk stratification beyond the traditional binary diagnosis of preeclampsia.

While the discriminatory performance of the sFlt-1/PlGF ratio alone was moderate, its value as part of a phenotype-based framework warrants further validation. A shift toward phenotype-oriented classification may contribute to more individualized monitoring and management strategies in preeclampsia.

Further prospective studies are warranted to validate phenotype-based models and to explore their role in precision obstetrics and personalized perinatal care.

## Figures and Tables

**Table 1:** Baseline demographic and obstetric characteristics of the study cohort.

Characteristic	Overall (*n* = 320)	Preeclampsia (*n* = 68)	No Preeclampsia (*n* = 252)	*p*-Value
Maternal age (years), mean ± SD	31.6 ± 5.9	31.1 ± 7.4	31.8 ± 5.4	0.621
Primigravida (Gesta = 1), *n* (%)	115 (35.9)	34 (50.0)	81 (32.1)	0.010
Gestational age at delivery (weeks), mean ± SD	37.6 ± 2.3	35.8 ± 3.4	38.1 ± 1.6	<0.001
Gestational diabetes, *n* (%)	12 (3.8)	2 (2.9)	10 (4.0)	1.000
Gestational hypertension, *n* (%)	27 (8.4)	2 (2.9)	25 (9.9)	0.084
NICU admission, *n* (%)	7 (2.2)	4 (5.9)	3 (1.2)	0.039

Values are presented as mean ± standard deviation (SD) for continuous variables and number (percentage) for categorical variables. Comparisons between groups were performed using Student’s *t*-test for continuous variables and the chi-square or Fisher’s exact test for categorical variables, as appropriate. A *p*-value < 0.05 was considered statistically significant. NICU = neonatal intensive care unit.

**Table 2:** Clinical and laboratory characteristics.

Parameter	Overall	Preeclampsia	No Preeclampsia	*p*-Value
Platelets (×10^3^/µL)	224.50 [188.15, 274.70]	214.50 [181.60, 270.58]	227.00 [189.82, 274.70]	0.151
WBC (×10^3^/µL)	11.71 [9.85, 13.60]	11.85 [9.65, 13.74]	11.68 [9.87, 13.56]	0.878
Fibrinogen (mg/dL)	519.00 [458.00, 590.00]	509.00 [457.75, 589.00]	524.00 [462.00, 597.00]	0.493
AST (U/L)	16.00 [14.00, 21.00]	16.00 [14.00, 21.25]	16.00 [14.00, 21.00]	0.709
ALT (U/L)	15.00 [12.00, 20.00]	19.00 [12.00, 28.50]	15.00 [12.00, 18.25]	0.001
Creatinine (mg/dL)	0.61 [0.57, 0.66]	0.62 [0.58, 0.68]	0.61 [0.56, 0.66]	0.243
Uric acid (mg/dL)	4.30 [3.60, 4.90]	4.40 [3.77, 5.05]	4.20 [3.50, 4.90]	0.106
Proteinuria	10.00 [0.00, 50.00]	55.00 [50.00, 100.00]	10.00 [0.00, 10.00]	<0.001
sFlt-1/PlGF ratio	28.70 [26.20, 53.82]	56.55 [46.10, 68.05]	27.40 [26.20, 36.35]	<0.001

Normality was assessed using the Shapiro–Wilk test. As most laboratory variables were non-normally distributed, results are reported as median [IQR] and compared using the Mann–Whitney U test.

**Table 3:** Perinatal outcomes according to preeclampsia phenotype.

Outcome	Hematologic (*n* = 27)	Hepatic (*n* = 13)	Renal (*n* = 28)	*p*-Value
Gestational age at delivery (weeks), mean ± SD	36.9 ± 2.8	35.9 ± 2.8	34.6 ± 3.9	0.070
Birth weight (g), mean ± SD	3007 ± 846	2633 ± 812	2196 ± 1011	0.006
NICU admission, *n* (%)	0 (0.0%)	1 (7.7%)	3 (10.7%)	0.229
Apgar score at 1 min, mean ± SD	8.0 ± 2.1	7.8 ± 2.5	6.6 ± 2.9	0.027

Normality was assessed prior to analysis. Continuous variables were compared using one-way ANOVA, while categorical variables were analyzed using the chi-square or Fisher’s exact test, as appropriate.

## Data Availability

The raw data supporting the conclusions of this article will be made available by the authors upon request.

## References

[1] Martini C., Saeed Z., Simeone P., Palma S., Ricci M., Arata A., Sorella A., Liani R., Ricci F., D’Antonio F. (2025). Preeclampsia: Insights into pathophysiological mechanisms and preventive strategies. Am. J. Prev. Cardiol..

[2] Kutllovci Hasani K., Ajeti N., Goswami N. (2025). Understanding Preeclampsia: Cardiovascular Pathophysiology, Histopathological Insights and Molecular Biomarkers. Med. Sci..

[3] Phipps E.A., Thadhani R., Benzing T., Karumanchi S.A. (2019). Pre-eclampsia: Pathogenesis, novel diagnostics and therapies. Nat. Rev. Nephrol..

[4] Papapanagiotou A., Daskalaki M.A., Gargalionis A.N., Margoni A., Domali A., Daskalakis G., Papavassiliou A.G. (2025). The Role of Angiogenetic Factors in Preeclampsia. Int. J. Mol. Sci..

[5] Foidart J.M., Schaaps J.P., Chantraine F., Munaut C., Lorquet S. (2009). Dysregulation of anti-angiogenic agents (sFlt-1, PLGF, and sEndoglin) in preeclampsia—A step forward but not the definitive answer. J. Reprod. Immunol..

[6] Ohkuchi A., Saito S., Yamamoto T., Minakami H., Masuyama H., Kumasawa K., Yoshimatsu J., Nagamatsu T., Dietl A., Grill S. (2021). Short-term prediction of preeclampsia using the sFlt-1/PlGF ratio: A subanalysis of pregnant Japanese women from the PROGNOSIS Asia study. Hypertens. Res..

[7] Nikuei P., Rajaei M., Roozbeh N., Mohseni F., Poordarvishi F., Azad M., Haidari S. (2020). Diagnostic accuracy of sFlt1/PlGF ratio as a marker for preeclampsia. BMC Pregnancy Childbirth.

[8] Hodel M., Blank P.R., Marty P., Lapaire O. (2019). sFlt-1/PlGF Ratio as a Predictive Marker in Women with Suspected Preeclampsia: An Economic Evaluation from a Swiss Perspective. Dis. Markers.

[9] Gyselaers W. (2020). Preeclampsia Is a Syndrome with a Cascade of Pathophysiologic Events. J. Clin. Med..

[10] Chiang Y.T., Seow K.M., Chen K.H. (2024). The Pathophysiological, Genetic, and Hormonal Changes in Preeclampsia: A Systematic Review of the Molecular Mechanisms. Int. J. Mol. Sci..

[11] Andronikidi P.E., Orovou E., Mavrigiannaki E., Athanasiadou V., Tzitiridou-Chatzopoulou M., Iatrakis G., Grapsa E. (2024). Placental and Renal Pathways Underlying Pre-Eclampsia. Int. J. Mol. Sci..

[12] Burwick R.M., Java A., Regal J.F. (2025). The role of complement in normal pregnancy and preeclampsia. Front. Immunol..

[13] Wang R.C., Wang Z. (2023). Precision Medicine: Disease Subtyping and Tailored Treatment. Cancers.

[14] Caillon H., Tardif C., Dumontet E., Winer N., Masson D. (2018). Evaluation of sFlt-1/PlGF Ratio for Predicting and Improving Clinical Management of Pre-eclampsia: Experience in a Specialized Perinatal Care Center. Ann. Lab. Med..

[15] Lecarpentier E., Tsatsaris V. (2016). Angiogenic balance (sFlt-1/PlGF) and preeclampsia. Ann. Endocrinol..

[16] Karpova N.S., Dmitrenko O.P., Budykina T.S. (2023). Literature Review: The sFlt1/PlGF Ratio and Pregestational Maternal Comorbidities: New Risk Factors to Predict Pre-Eclampsia. Int. J. Mol. Sci..

[17] Benton S.J., Leavey K., Grynspan D., Cox B.J., Bainbridge S.A. (2018). The clinical heterogeneity of preeclampsia is related to both placental gene expression and placental histopathology. Am. J. Obs. Gynecol..

[18] Chaiworapongsa T., Romero R., Gomez-Lopez N., Suksai M., Gallo D.M., Jung E., Berry S.M., Awonuga A., Tarca A.L., Bryant D.R. (2024). Preeclampsia at term: Evidence of disease heterogeneity based on the profile of circulating cytokines and angiogenic factors. Am. J. Obstet. Gynecol..

[19] Schallenberg S., Dernbach G., Ruane S., Jurmeister P., Böhm C., Standvoss K., Ghosh S., Frentsch M., Dragomir M.P., Keyl P.G. (2025). AI-powered spatial cell phenomics enhances risk stratification in non-small cell lung cancer. Nat. Commun..

[20] Shaon M.A., Farhana F.Z., Sikder R., Shrestha K., Haque F., Tantu M.T., Bin Manjur O.H., Aktar S., Koo K.M., Ross A.G. (2025). Preeclampsia Diagnostics and Therapeutics: Advances, Challenges, and Prospects in Nanoscale Exosome-Based Clinical Translation. ACS Nano Med..

[21] Garunkštienė R., Vaitkevičienė R., Paulavičienė I., Drazdienė N., Čerkauskienė R. (2018). Acute kidney injury in an extremely low birth weight infant with nephrolithiasis: A case report. Acta Medica Litu..

[22] Murvai V.-R., Galiș R., Macrea C.-M., Tărău-Copos A.-F., Goman M.D., Ghitea T.C., Huniadi A. (2025). The Impact of Thrombophilia on Maternal and Neonatal Outcomes: A Multisystem Analysis of Clinical, Hematological, and Metabolic Parameters. J. Clin. Med..

[23] Sekulovski M., Mileva N., Chervenkov L., Peshevska-Sekulovska M., Vasilev G.V., Vasilev G.H., Miteva D., Tomov L., Lazova S., Gulinac M. (2023). Endothelial Dysfunction and Pregnant COVID-19 Patients with Thrombophilia: A Narrative Review. Biomedicines.

[24] Murvai V.R., Radu C.-M., Galiș R., Ghitea T.C., Tătaru-Copos A.-F., Vesa A.-A., Huniadi A. (2025). The Relationship Between Thrombophilia and Modifications in First-Trimester Prenatal Screening Markers. Medicina.

[25] Toma A.I., Dima V.A.-O., Rusu L., Nemeș A.F., Gonț B.F., Arghirescu A., Necula A., Fieraru A., Stoiciu R., Andrășoaie L. (2024). Cerebral Ultrasound at Term-Equivalent Age: Correlations with Neuro-Motor Outcomes at 12-24 Months Corrected Age. Children.

[26] Martin-Alonso R., de Paco Matallana C., Valiño N., Chaveeva P., Dagklis T., Siargkas A., Wright A., Camacho M., Rolle V., Santacruz B. (2025). Prediction of Small for Gestational Age and Growth-Restricted Neonates at 35 to 36 Weeks of Gestation: A Multicenter Cohort Study. Medicina.

[27] Libretti A., Valsecchi L., Zerbini G., Remorgida V., Candiani M. (2023). Maternal plasma markers in intrauterine growth restriction and small for gestational age complicated pregnancy: The role of sFlt-1/PlGF. Minerva Obstet. Gynecol..

[28] Trif P., Sava C., Mudura D., Kramer B.W., Galiș R., Ognean M.L., Iuhas A., Jurca C.M. (2025). Seasonal Patterns of Preterm Birth During the COVID-19 Pandemic: A Retrospective Cohort Study in Romania. Medicina.

[29] Ng K.W., Chaturvedi N., Coté G.L., Fisher S.A., Mabbott S. (2024). Biomarkers and point of care screening approaches for the management of preeclampsia. Commun. Med..

[30] Garrido-Giménez C., Cruz-Lemini M., Álvarez F.V., Nan M.N., Carretero F., Fernández-Oliva A., Mora J., Sánchez-García O., García-Osuna Á., Alijotas-Reig J. (2023). Predictive Model for Preeclampsia Combining sFlt-1, PlGF, NT-proBNP, and Uric Acid as Biomarkers. J. Clin. Med..

[31] Maynard S., Epstein F.H., Karumanchi S.A. (2008). Preeclampsia and angiogenic imbalance. Annu. Rev. Med..

[32] Nasir M., Asif A.B., Waheed M., Irfan J., Khan Q.U., Waseem A. (2025). Exploring Preeclampsia: A Comprehensive Overview. Discoveries.

[33] Kariori M., Katsi V., Tsioufis C. (2025). Late vs. Early Preeclampsia. Int. J. Mol. Sci..

[34] Bdolah Y., Sukhatme V.P., Karumanchi S.A. (2004). Angiogenic imbalance in the pathophysiology of preeclampsia: Newer insights. Semin. Nephrol..

[35] Vidaeff A.C., Saade G.R., Sibai B.M. (2021). Preeclampsia: The need for a biological definition and diagnosis. Am. J. Perinatol..

